# Occupational Therapy in Mental Health via Telehealth during the COVID-19 Pandemic

**DOI:** 10.3390/ijerph18137138

**Published:** 2021-07-03

**Authors:** Antonio José Sánchez-Guarnido, Esther Domínguez-Macías, José Antonio Garrido-Cervera, Roberto González-Casares, Silvia Marí-Boned, Águeda Represa-Martínez, Carlos Herruzo

**Affiliations:** 1 Department of Mental Health, Hospital Santa Ana, 18009 Granada, Spain; antonioj.sanchez.guarnido.sspa@juntadeandalucia.es (A.J.S.-G.); roberto.gonazalez.casares.sspa@juntadeandalucia.es (R.G.-C.); 2 Department of Mental Health, Hospital de la Axarquía, Vélez-Málaga, 29700 Málaga, Spain; esdoma82@hotmail.com; 3 Department of Mental Health Hospital Virgen de la Victoria/University of Málaga, 29010 Málaga, Spain; josegarrido79@hotmail.com; 4 Department of Mental Health Hospital Can Misses, Eivissa, 07800 Illes Balears, Spain; smari@asef.es (S.M.-B.); aguedarepresa@gmail.com (Á.R.-M.); 5 Department of Psychology, University of Córdoba, 14071 Córdoba, Spain

**Keywords:** COVID-19, pandemic, occupational therapy, telehealth, mental health

## Abstract

The COVID-19 pandemic has brought about changes in mental health occupational therapy. Research into these changes and the associated risks of relapse is insufficient. To explore the changes that have taken place in forms of occupational intervention (face-to-face and online) during the pandemic, and to analyze their association with subsequent relapses, a multicenter retrospective cohort study was carried out of 270 patients with mental disorder diagnoses under follow-up in day hospitals during 2020. Our results show that the frequency of face-to-face occupational therapy interventions decreased during lockdown and subsequently recovered. Interventions via telehealth increased during lockdown and have since been continued to a greater extent than before lockdown. Patients who received occupational intervention via telehealth relapsed less in the following six months (10.7% vs. 26.3%; χ^2^ = 10.372; *p* = 0.001), especially those who received intervention via videoconferencing (4.2% vs. 22%; χ^2^ = 5.718; *p* = 0.017). In conclusion, lockdown subsequent to the COVID-19 outbreak led to a reduction in face-to-face occupational therapy interventions, putting people with prior mental disorders at risk, while the implementation of telehealth tools helped reduce relapses.

## 1. Introduction

COVID-19 is a pathology with mainly physical symptoms that can also affect the occupational balance and mental health of the population, especially vulnerable people like those who have previously suffered from mental disorders [1].

With regard to its physical consequences, the disease has been shown to cause pulmonary, cardiac, and muscular problems and neurological manifestations that directly affect mobility and the performance of daily activities, both basic and instrumental, in people undergoing recovery [2,3]. The COVID-19 crisis and the home confinement to which the population was subjected during the lockdown period also had a significant impact on occupational activity in all areas of daily life [4]. This impact has resulted in major changes in behavioral patterns: interruption of daily routines and occupations, modification of habits, adoption of new and/or previously unperformed roles, and the impossibility of executing the rituals that form part of the individual’s occupational identity [5]. Social isolation has been even more pronounced in vulnerable people such as those with some type of disability [6] who may have more difficulties in developing adaptive responses to the occupational imbalance caused by the period of confinement [4].

Occupational therapy plays a fundamental role in this scenario, both in the occupational care of people who have been seriously affected by the pandemic and in the maintenance and recovery of activities of daily living [5,7]. The impact that COVID-19 may have had on occupational therapy for people with previous mental disorders is especially important. Evidence-based clinical practice guidelines recommend offering occupational therapy to people with severe mental disorders (SMDs) during both the acute phase and the recovery phase [8], as this has proven useful in improving social functioning and reducing the number of readmissions [9].

Despite such recommendations, however, the COVID-19 pandemic has increased the risk of a reduction in the provision of mental health services in general and, by extension, of occupational therapy services, since in this period, face-to-face interventions have increased the risk that the illness may be transmitted to patients and professionals [10]. To mitigate as much as possible the consequences of the lack of direct personal contact, telephone and videoconferencing consultations were implemented as an alternative to face-to-face care. Previous experience with these types of mental health interventions has generally been favorable in terms of patient acceptance and satisfaction, feasibility, and efficacy, without negatively affecting the therapeutic relationship [11]. However, relatively little research has been carried out into the effectiveness of telepsychiatry for severe mental disorders. The use of digital technology-based therapy is also known to be lower for people with psychosis than for the general population [12] and in addition, it should be noted that there may be processing affected by dysfunction of the Dorso-Lateral Prefrontal Cortex [13,14]. Nevertheless, the limited data available seems to indicate that the use of modalities like telephone, internet, and videoconferencing is feasible in patients with schizophrenia and other mental disorders. Preliminary evidence even suggests that such modalities actually improve patients, but more research is needed [15]. In addition to videoconferencing, other text-messaging or social networking applications may also enhance attention and provide support [10].

Occupational therapists have had to adapt to these new forms of care, and there is some evidence to affirm that telehealth in the field of occupational therapy yields benefits. There is also a high level of satisfaction on the part of the users who receive it [16]. In the field of mental health, occupational therapists can therefore use telehealth to work proactively to prevent the onset of symptoms and enhance the recovery model, allowing equal access and better opportunities for all those who have mental health problems, their families, and their closest social networks [17].

Finally, and in summary, research into COVID-19 and its physical consequences should not obviate the need to study its effects at the occupational level and on mental health, especially for the most vulnerable people, such as those with a previous history of mental problems. The measures taken to control the pandemic in its first few months involved changes in occupational therapy services in mental health and generated a risk of relapse for people with previous mental disorders. With this in mind, the objectives of the present study were to explore what changes have occurred in the care received via both face-to-face and telehealth occupational therapy, and the relationship between those changes and relapses.

## 2. Materials and Methods

### 2.1. Design

Multicenter retrospective cohort study.

### 2.2. Participants

The study addressed people diagnosed with a mental disorder who had been under follow-up in mental health day hospitals (MHDHs) during the year 2020. The participants, which included both men and women, were all over 18 years of age. The point of departure was a definition of severe mental disorder (SMD) that included the patient’s psychopathological diagnosis and a need for high intensity treatment.

A sample size of 272 patients was calculated for a confidence level of 95%, a statistical power of 80%, and an estimated relapse rate of 30% in the control group and 20% in the intervention group, with possible losses of 15%. Using stratified sampling by region to facilitate generalization to the Spanish population, fifteen MHDHs of the Spanish National Health System took part in the study. Data were collected from 270 patients, of whom 120 were men and 150 women, aged between 18 and 67 years (average age 39.90). The most frequent diagnoses were severe mental disorders like schizophrenia and other psychotic disorders (30.4%), personality disorder (27.8%), and bipolar disorder (10.4%). Most of the sample had received primary (35.6%) or secondary (41.5%) education, while 14.8% had studied at the university level. The majority lived with their family of origin (28.9%), in their own family home (28.9%), or alone (17%). A total of 29.3% were retired, 26.3% were unemployed, 20% were temporarily disabled, and 16.7% were working (see Table 1).

### 2.3. Procedure

Data were collected retrospectively during the months of October and November 2020 by collaborators in each MSDSH, from clinical histories and interviews with the patients.

Informed consent was requested from all participants. A database was designed to which only the researchers had access, and clinical data were processed without patient identification data.

### 2.4. Variables

The sociodemographic variables studied were age, sex, household composition, employment status, and level of education.

To analyze how therapy interventions changed during the pandemic, we used the moment of analysis in relation to the beginning of the first wave and the first lockdown in Spain as an independent variable, establishing three two-month observation periods: the period prior to lockdown (from 16 January to 15 March), the period of strict lockdown (from 16 March to 15 May), and the period of de-escalation (from 16 May to 15 July). The dependent variables were the occupational therapy interventions received by patients in each period, which were collected from the clinical history and coded dichotomously (i.e., received or not received). Received occupational therapy interventions were included respectively, according to whether they were received in person, by telephone, by videoconference, or by other telematic means (Facebook, messaging, e-mail, or blog).

To study the relationship between these changes in intervention and relapse, having received occupational intervention via telehealth during the period of lockdown was taken as an independent variable. This was presented as a dichotomous variable. For this second objective, the percentage of patients who relapsed (relapse being defined as full hospitalization) at two, four, and six months was used as the main dependent variable. We also used the mean difference in the number of urgent mental health consultations at 2, 4, and 6 months as a secondary, dependent variable.

Finally, three modes of telehealth (telephone, video-call, and other telematic means) were compared in terms of the percentage of admissions to inpatient units at six months.

With respect to occupational interventions via telehealth, the telephone was used mainly for the individual follow-up of routines and habits, vocational interventions, and support for activities of daily living. Video calls were group-based, with an open framework in which the meaningful occupation of free time was monitored. With regard to other telematic media, Facebook and blogs were used to make proposals and provide material to support the implementation of activities (physical activity, cognitive stimulation, leisure activities, and vocational orientation) during the lockdown period. Finally, mobile messaging and e-mail were used as alternative means of communication for the individual monitoring of occupational activity.

### 2.5. Data Analysis

The data were analyzed statistically using the IBM SPSS V.21.0 program. (IBM Corp., Armonk, NY, USA) The level of statistical significance for this study was *p* < 0.05.

The results of categorical variables were expressed as percentages and those of quantitative variables as mean and standard deviation.

For between-group analyses, Chi-square was used for associations between categorical variables and Student’s t-test was used to compare groups of quantitative variables.

McNemar’s test was used to analyze significant changes between the measurements of dichotomous variables of the same individuals at different times in the study.

The project was approved by the research ethics committee of the different participating hospitals and the study complied at all times with the principles of the Helsinki Declaration. Data confidentiality was respected in accordance with current European Union (EU) legislation.

## 3. Results

### 3.1. Analysis of Changes in Occupational Intervention

Table 2 compares different modes of occupational therapy interventions in the different periods according to the medium used. The percentage of patients receiving occupational therapy face-to-face in the different periods went from 45.9% before lockdown, to 7% during lockdown, and then returned to 41.9% after lockdown. These differences are statistically significant between pre-lockdown and lockdown (χ2 = 92.444; *p* < 0.001), and between lockdown and post/lockdown (χ2 = 88.255; *p* < 0.001). They are not, however, significant between pre-lockdown and post-lockdown (χ2 = 1.754; *p* = 0.185).

The percentage of patients receiving telephone interventions went from 1.5% before lockdown to 20.7% during lockdown, and then dropped to 6.7% in the post-lockdown period. These differences are statistically significant between the pre-lockdown and lockdown periods (χ^2^ = 50.019; *p* < 0.001), between the pre-lockdown and post-lockdown periods (*p* < 0.001), and also between lockdown and the post-lockdown periods (χ^2^ = 27.38; *p* < 0.001).

Occupational therapy interventions performed by videoconference were non-existent before lockdown but were received by 8.9% of patients during lockdown. This percentage dropped to 2.2% in the post-lockdown period. Again, these differences are statistically significant between the pre-lockdown and lockdown periods (*p* < 0.001), between lockdown and the post-lockdown periods (*p* < 0.001), and between the pre-lockdown and post-lockdown periods (*p* = 0.031).

Occupational therapy interventions performed using other telematic platforms were received by 2.2% of patients before lockdown, by 28.5% of patients during lockdown, and by 19.6% of patients in the post-lockdown period. These differences are statistically significant between the pre-lockdown and lockdown periods (χ^2^ = 67.123; *p* < 0.001), between the lockdown and de-escalation periods (χ^2^ = 13.921 *p* < 0.001), and between the pre-lockdown and post-lockdown periods (χ^2^ = 39.925; *p* < 0.001).

### 3.2. Analysis of Occupational Intervention via Telehealth and Relapse

#### 3.2.1. Description of the Comparison Groups

Table 1 describes the sociodemographic characteristics and Table 3 describes the clinical characteristics of the group that received occupational therapy via telehealth and the group that did not. There were no differences between the groups in terms of diagnosis (χ^2^ = 2.911; *p* = 0.573), adherence to treatment (χ^2^ = 0.033; *p* = 0.856), age (t = 0.410; *p* = 0.682), sex (χ^2^ = 0.314; *p* = 0.575), composition of the households in which the patients lived (χ^2^ = 8.204; *p* = 0.224), or levels of education (χ^2^ = 4.196; *p* = 0.123). There were significant differences in work activity: in the group that received occupational therapy intervention via telehealth, only 2.9% were working compared to 25.1% in the group that did not receive such intervention (χ^2^ = 37.936; *p* < 0.001).

#### 3.2.2. Comparison of Clinical Results

Table 1 also shows the percentages for hospital admissions occurring during the months following lockdown in the two groups. At two months, the percentage of patients admitted was significantly lower in the telehealth intervention group compared to the non-intervention group. These differences were maintained at four (6.8% vs. 18%; χ^2^ = 7.336; *p* = 0.007) and at six months (10.7% vs. 26.3%; χ^2^ = 10.372; *p* = 0.001).

In relation to emergency consultations, the mean number at two months was 0.215 times lower in patients who received occupational intervention via telehealth but was not significant (t = 1.598; *p* = 0.111). At four months, this difference increased to 0.57409, becoming statistically significant (t = 2.341; *p* = 0.020). At six months, it rose to 0.71316 (t = 1.896; *p* = 0.059), although again without statistical significance.

#### 3.2.3. Comparison of Relapses According to the Medium Used

Table 4 shows the percentages of hospital admissions according to the different intervention subtypes. Among patients who received intervention by telephone, admissions were lower but not statistically significant (12.5% vs 22.4%; RR = 0.56; χ^2^ = 2.946; *p* = 0.086). Differences were significant for videoconference interventions compared to non-intervention but also to other telematic intervention.

## 4. Discussion

As expected, analysis of occupational therapy interventions performed during the pandemic showed a decrease in face-to-face care and an increase in telehealth interventions during the lockdown period. After lockdown, most face-to-face mental health care was restored and remote interventions were reduced, although their percentage remained higher than in the previous period. This could be related to the fact that the return to face-to-face activity in mental health facilities was conditioned in most cases by capacity restrictions, with telematic care being maintained as a complementary tool. However, it may also be interpreted in terms of the perceived usefulness of telematic tools during the lockdown period. Other researchers have also looked at how mental health care underwent changes during the COVID-19 pandemic and how many countries resorted to telehealth, although the implementation of remote interventions and their results were found to have been mixed [1]. Recent publications suggest that teleconsultations have been a valuable resource but cannot completely replace face-to-face consultations [18], and that there have been difficulties in implementing telehealth services for mental patients [19].

According to our data, the channels most frequently used by occupational therapists during lockdown were social networks, blogs, messaging, and e-mail. This group of tools has continued to be used during the post-lockdown period at much higher levels than in the pre-pandemic stage. These results are in line with those obtained in other studies, highlighting the feasibility and impact of the use of new technologies in clinical practice [9].

The second most commonly used mode of care provision in our sample was by telephone, possibly related to its greater availability and to lower resistance to its use among both professionals and patients. Here, the recommendations are that telephone consultations can be used to facilitate participation and follow-up for those who lack access to digital technology or the skills or confidence to use videoconferencing platforms [20]. The use of this type of intervention also subsequently remained above pre-lockdown levels.

Mental health occupational therapists did not resort to videoconferencing until lockdown, with interventions of this type still occasionally being carried out in the post-lockdown period. We found similar results in tele-rehabilitation studies, highlighting the positive impact, on patients and on health in general, of using tools that can help develop people’s functional performance [21]. The fact that videoconferencing has been used to a lesser extent than, for example, the telephone, may be due to the lack of appropriate resources in our devices, as we often lack secure applications, correctly configured computers, or good internet connections.

Our results also suggest a link between occupational interventions via telehealth and a reduction in relapses. This is consistent with a growing body of research which, even prior to the pandemic, had already indicated that intervention via internet and cell phones upholds the benefits of treatment and aids relapse prevention in different mental disorders [22], highlighting the applicability of telemedicine interventions in the tertiary prevention of chronic or recurrent mental disorders [23]. In addition, our data showed that access to emergency services among patients receiving telematic occupational therapy interventions was also lower compared to those who did not receive such interventions. Previous research has already shown that continuity of care is essential to avoid decompensation and is reflected in a lower use of emergency services [24].

Comparison of the different subtypes of telehealth intervention in relation to relapse demonstrated the greater effectiveness of online interventions, especially videoconferencing. Thus, although it is well known that psychiatric disorders such as schizophrenia and bipolar disorders are associated with impaired processing of emotional faces [25], in most of the studies reviewed, the telematic interventions in the field of mental health that proved to be the most effective in relapse prevention were carried out using applications that included face-to-face contact with a therapist. Improvements in treatment adherence, social functioning, and quality of life have also been found through videoconferencing, even in some cases, similar to those observed in the face-to-face intervention [26]. These observed improvements in videoconferencing over other telematic interventions may be related to greater ease in maintaining key aspects of the intervention such as the therapeutic alliance [27], which plays a crucial role in the outcome of therapy [28]. In addition, the use of mobile applications, including guidelines for the maintenance of routines or activities of daily living, also yields positive results in terms of maintaining outpatient autonomy [22].

Our study enabled us to analyze the changes that took place in mental health occupational therapy interventions during the pandemic, a subject insufficiently addressed in the scientific literature. The use of day hospitals as participating centers ensured good data reliability, with high quality records. The six-month follow-up made it possible to analyze changes over time and how they related to subsequent relapses. Furthermore, the fact that this was a multicenter study also favored a greater generalizability of our conclusions. One of the study’s limitations was that it was an observational, retrospective study, and may therefore have included some biases, both in relation to variables that may have influenced the results but were not studied, and regarding the data collected (although objective variables based on medical records were used to reduce such biases). Neither did the study allow any comparisons to be made between telematic interventions and face-to-face interventions, because in the period studied there were hardly any face-to-face interventions and, furthermore, those that did take place focused on patients in crisis and were not therefore comparable. Likewise, the interventions carried out using different telehealth media could have been different and are therefore difficult to compare.

Further research is needed in this area, using other measures based on performance and recovery models. It would also be interesting to monitor the use of different telematic tools over time to see if they remain in the service portfolio when the pandemic is over. The potential benefits of telematic interventions in non-pandemic scenarios should also be further explored. This would require experimental studies standardizing interventions by occupational area.

## 5. Conclusions

The lockdown subsequent to the COVID-19 pandemic led to a reduction in face-to-face occupational therapy interventions and increased the risk of worsening mental health in people with previous disorders. Fortunately, the pandemic also provided an opportunity to extend the use of telehealth tools in the occupational therapy approach to mental health. The use of these tools has helped to reduce relapses in mental patients. Unfortunately, however, not all patients have had the opportunity to benefit from telematic therapy. In the future, we should not allow occupational interventions for patients with mental disorders to be interrupted again. If face-to-face care cannot be offered, telehealth interventions should be guaranteed, whenever possible facilitating contact by video call rather than by telephone. More research is still needed, both into the impact of the COVID-19 pandemic on occupational therapy interventions and into the effectiveness of telehealth beyond the pandemic context.

## Figures and Tables

**Table 1:** Sociodemographic characteristics.

	Total	Telematic OT Intervention Group	Non-Intervention Group	*p*
Age; mean (SD)	39.90 (11.81)	40.13 (11.94)	39.52 (11.66)	t = 0.410 <0.682
**Sex**				χ^2^ = 0.314 <0.575
Men	120 (44.4%)	72 (43.1%)	48 (46.6%)	
Women	150 (55.6%)	95 (56.9%)	55 (53.4%)	
Total	270 (100%)	167 (100%)	103 (100%)	
**Household composition**				χ^2^ = 8.204 <0.224
Full family of origin (parents with orwithout siblings)	78 (18.9%)	45 (26.9%)	33 (32%)	
Own family household (married, cohabiting, and/or with children)	78 (18.9%)	45 (26.9%)	33 (32%)	
Horizontal (with friends or siblings)	16 (5.9%)	12 (7.2%)	4 (3.9%)	
Single parent (single parent with or without siblings)	37 (13.7%)	20 (12%)	17 (16.5%)	
Other	7 (2.6%)	5 (3%)	2 (1.9%)	
Single-person	46 (17 %)	33 (19.8%)	13 (12.6%)	
Institutionally supervised housing (sheltered housing, group home. etc.)	8 (3%)	7 (4.2%)	1 (1%)	
Total	270 (100%)	167 (100%)	103 (100%)	

OT: Occupational Therapy.

**Table 2:** Time periods in the pandemic and changes in care received.

	Clinics			Comparison		
	% Patients Pre-Lockdown (January 16–March 15)	% Patients Lockdown (March 16–May 15)	% Patients Post-Lockdown (May 16–July 15)	Before and during Lockdown	Before and after Lockdown	During and after Lockdown
Changes in the care received						
On-site occupational therapy	124 (45.9%)		113 (41.9%)	χ^2^ = 92.444 *p =* 0.001	χ^2^ = 88.255 *p =* 0.001	χ^2^ =1.754 *p =* 0.185
Telephone occupational therapy	4 (1.5%)	56 (20.7%)	18 (6.7%)	χ^2^ = 50.019 *p =* 0.001	*p =* 0.001	χ^2^ = 27.38 *p =* 0.001
Occupational therapy by videoconference	0 (0%)	24 (8.9%)	6 (2.2%)	*p =* 0.001	*p =* 0.250	*p =* 0.001
Other telematic interventions	6 (2.2%)	77 (28.5%)	53 (19.6%)	χ^2^ = 67.123 *p =* 0.001	χ^2^ = 13.921 *p =* 0.001	χ^2^ = 39.925 *p =* 0.001

**Table 3:** Clinical characteristics of the group that received occupational therapy.

	Total	Telematic OT Intervention Group	Non-Intervention Group	*p*
***Diagnosis***				
Schizophrenia or other psychotic disorders	82 (30.4 %)	51 (30.5%) * [25%]	31 (30.1%)	χ^2^ = 2.911
Personality disorder	75 (27.8%)	43 (25.7%) * [41.6%]	32 (31.1%)	*p* = 0.573
Bipolar disorder	28 (10.4%)	18 (10.8%) * [4.2%]	10 (9.7%)	
Depressive disorder	26 (9.6%)	14 (8.4%) * [16.7%]	12 (11.7%)	
Other	59 (21.9%)	41 (24.6%) * [12.5%]	18 (17.5%)	
Total	270 (100%)	167 (100%)	103 (100%)	
**Treatment adherence**	232 (85.9%)	88 (85.4%)	144 (86.2%)	χ^2^ = 0.033
				*p* = 0.856
**Full hospitalization during the period of evaluation**				
Admissions at 2 months	18 (6.7%)	3 (2.9%)	15 (9.0%)	χ^2^ = 4.225 *p* = 0.040
Admissions at 4 months	37 (13.7%)	7 (6.8%)	30 (18.0%)	χ^2^ = 7.336 *p* = 0.007
Admissions at 6 months	55 (20.4%)	11 (10.7%)	44 (26.3%)	χ^2^ = 10.372 *p* = 0.001
		Average differences		
**Consultation with emergency mental health services via any channel**				
Emergency consultations at 2 months		0.215		t = 1.598 *p* = 0.111
Emergency consultations at 4 months		0.574		t = 2.341 *p* = 0.020
Emergency consultations at 6 months		0.713		t = 1.896 *p* = 0.059

* Video-conferencing intervention.

**Table 4:** Risk of relapse with hospital admission as a function of the different telematics interventions.

	Relapses with Intervention	Relapses Without Intervention	Relative Risk of Elapse	Chi-Square	*p*
Intervention by telephone	12.5	22.4	0.56	2.946	*p* = 0.086
Intervention by videoconference	4.2		0.19	5.718	*p* = 0.017
Intervention by other telematic means	14.4	21.6	0.66	7.327	*p* = 0.007

## Data Availability

The data presented in this study are available on request from the corresponding author. The data are not publicly available because they are part of an ongoing project.
